# Radiofrequency ablation versus laparoscopic hepatectomy for hepatocellular carcinoma: a systematic review and meta-analysis

**DOI:** 10.1186/s12957-024-03473-8

**Published:** 2024-07-24

**Authors:** Chuang Jiang, Qingbo Feng, Zhihong Zhang, Zeyuan Qiang, Ao Du, Lin Xu, Jiaxin Li

**Affiliations:** 1 Division of Liver Surgery, Department of General Surgery and Laboratory of Liver Surgery, State Key Laboratory of Biotherapy and Collaborative Innovation Center of Biotherapy, West China Hospital, Sichuan University, Chengdu, China; 2 Department of General Surgery, Digestive Disease Hospital, Affiliated Hospital of Zunyi Medical University, Zunyi, Guizhou China; 3 Department of General Surgery, Dafang County People’s Hospital, Bijie, Guizhou Province 551600 China

**Keywords:** Hepatocellular carcinoma, Laparoscopic hepatectomy, Liver resection, Radiofrequency ablation, Meta-analysis

## Abstract

**Background:**

Although laparoscopic hepatectomy (LH) and radiofrequency ablation (RFA) are the 2 principal minimally invasive surgical approaches and the first line of treatments for patients with hepatocellular carcinoma (HCC). It is not clear which one has greater safety and efficacy. In this meta-analysis, we aim to compare the safety and effectiveness of LH versus RFA for patients with HCC, especially where perioperative and postoperative outcomes differrent.

**Methods:**

In PROSPERO, a meta-analysis with registration number CRD42021257575 was registered. Using an established search strategy, we systematically searched Web of Science, PubMed, and Embase to identify eligible studies before June 2023. Data on operative times, blood loss, length of stay, overall complications, overall survival (OS) and recurrence-free survival (RFS) were subjected to meta-analysis.

**Results:**

Overall, the present meta-analysis included 8 retrospective and 6 PSM studies comprising 1,848 patients (810 and 1,038 patients underwent LH and RFA). In this meta-analysis, neither LH nor RFA showed significant differences in 1-year and 3-year OS rate and 5-year RFS rate. Despite this, in comparison to the RFA group, LH resulted in significantly higher 1-year(*p*<0.0001) and 3-year RFS rate (*p* = 0.005), higher 5-year OS rate (*p* = 0.008), lower local recurrence rate (*p*<0.00001), longer length of stay(LOS) (*p*<0.0001), longer operative time(*p*<0.0001), more blood loss (*p*<0.0001), and higher rate of complications (*p*=0.001).

**Conclusions:**

Comparative studies indicate that LH seemed to provide better OS and lower local recurrence rate, but higher complication rate and longer hospitalization.

**Supplementary Information:**

The online version contains supplementary material available at 10.1186/s12957-024-03473-8.

## Introduction

As the prevalence of hepatocellular carcinoma (HCC) increases, it has become the third leading cause of cancer-related death worldwide [1]. At present, there are many treatments available for HCC, including hepatectomy, liver transplantation, TACE, RFA, microwave coagulation, molecular targeted drugs and radiotherapy [2–4]. Hepatectomy and liver transplantation are generally recognized as most effective methods for the treatment of HCC, but liver transplantation can’t be widely carried out due to the shortage of donor livers [5]. In recent years, minimally invasive surgery represented by laparoscopic hepatectomy (LH) and RFA has gradually become a new choice for the treatment of early-stage HCC. The use of LH and RFA is effective and a potentially curative treatment for early-stage HCC, and can provide a cure or prolong survival [6–8]. Laparotomy is often used for LH because of its complexity. Nevertheless, LH has become increasingly popular since Reich et al. reported it in 1991 [9]. Several literature reviews have affirmed RFA’s safety and efficacy, highlighting RFA’s advantages over open hepatectomy in terms of less blood loss, shorter operation times, and faster recovery times [10, 11]. And, few robust trials have compared the long-term oncological outcomes of LH with RFA and existing results are also not consistent. Although there has been very limited studies comparing surgical and oncological outcomes between LH and RFA for HCC [12, 13], some high-quality studies weren’t included in these reviews. In this study, we compared long-term outcomes and perioperative outcomes between LH and RFA for HCC.

## Methods

This meta-analysis was performed by the PRISMA guidelines and the protocol of this study was registered in PROSPERO(CRD42021257575).

### Literature search and study selection

Two independent researchers (CJ, QF performed a systematic search of PubMed, EMBASE, and Web of Science in accordance with PRISMA guidelines for the studies that provided comparisons between RFA(percutaneous or laparoscopic RFA) and LH for HCC [14]. The combinations of following terms were used: “radiofrequency ablation” or “RFA”, “laparoscopic”, “laparoscopic hepatectomy”, “liver resection”, or “minimally invasive”, “HCC”,“hepatocellular carcinoma” or “liver cancer”. Additional studies were gained by manually searching the references of eligible studies .

### Inclusion and exclusion criteria

A review and screening of all titles and abstracts of all submitted papers was conducted independently by two investigators (CJ and QF).

Inclusion criteria were: (1) Participants: patients with HCC; (2) Intervention: LH or RFA; (3) Study type: observational clinical studies, randomized controlled trials (RCTs), case-control studies; (4) at least one interested data has been reported.

Exclusion criteria were: (1) Expert opinions, editorials, abstracts, letters, and case reports ; (2) Studies without available data.

### Data extraction and quality assessment

Based on a unified datasheet, two reviewers(CJ, QF) independently extracted data and resolved disagreements by discussion .We extracted the following major data: first author, research design, publication year, country, sample size, age, tumor size, operative times, blood loss, hospitalization, incidence of complications, overall survival (OS) and recurrence-free survival (RFS). In this review, the Newcastle-Ottawa Scale (NOS) was used to evaluate the quality of studies included, with performance scores of 6 being regarded as high. [15]

### Statistical analysis

Review Manager 5.3 software was used to analyze dichotomous data using odds ratios (ORs), and weighted mean differences (MDs) and confidence intervals (95% CI) for continuous data. In order to extract OS and RFS data from Kaplan-Meier curves, the Engauge Digitizer v.4.1 software was used [16]. In studies that reported only medians and ranges, Hozo et al.‘s original method could be used to estimate the mean and standard deviation [17]. Begg’s funnel plot and Egger’s test were used to assess publication bias. The X^2^ test with I^2^ was used to quantify heterogeneity. When I2 < 50%, representing heterogeneity is low or moderate, a fixed-effects model (FEM) was adopted, while I^2^ ≥ 50%, (heterogeneity is high) a random-effects model (REM) was used.

## Results

### Literature search result and quality assessment

**Fig. 1:**
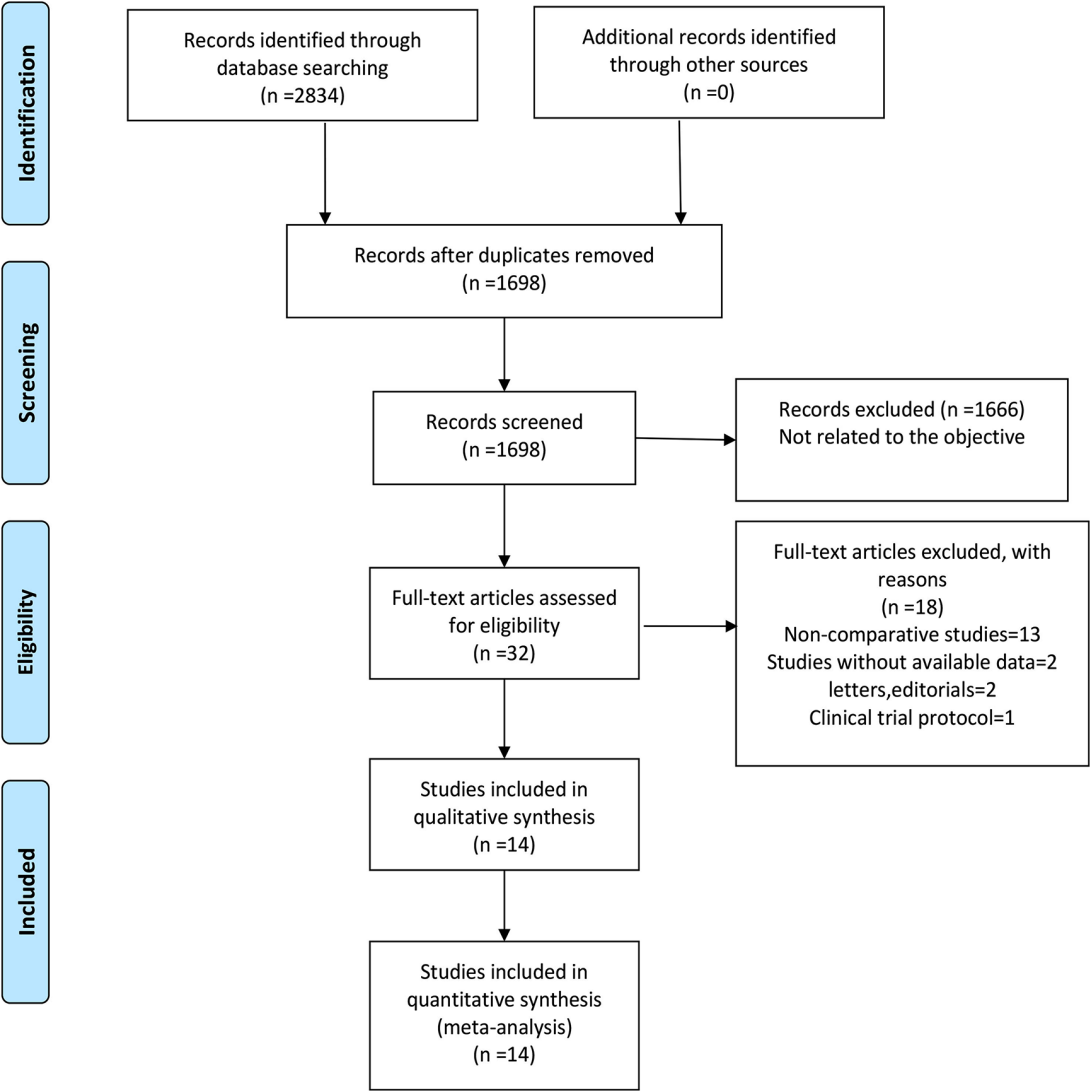
Flow chart of study identification and selection

Based on the various electronic databases, 2,834 relevant English publications were identified. The final analysis included 14 retrospective studies comparing LH and RFA in a total of 1,848 patients (810 received LH and 1,038 received RFA, respectively) [18–31]. Six of them are propensity-score matching (PSM) studies which can ensure the baseline data of patients were consistent [21, 22, 27–30]. We only use the data after PSM from six studies to minimize selection bias. Figure 1 summarizes the PRISMA flowchart for study selection. In Table 1, we summarize the general information and the NOS stars of all eligible studies.The results of using the Cochrane risk of bias tool to assess risk of bias is presented in Figure S1. All results of interest outcomes of this meta-analysis are given in Table 2.

**Table 1:** Study characteristics

Study	Type of study	Research time	Country	Patients		Age(years)		Gender(M/F)		NOS
				RFA	LH	RFA	LH	RFA	LH	
Casaccia-2015	Retrospective	2005–2010	Italy	24	26	61.48 ± 7.75	64.62 ± 9.51	20/4	17/9	8
Song-2015	Retrospective	2007–2013	China	78	78	48	48	70/8	70/8	8
Vitali-2015	Retrospective	1998–2012	France	60	45	67.3(47–83)	61.4(31–84)	52/8	30/15	8
Harada-2016	PSM	2008–2015	Japan	20	20	73 ± 9	74 ± 6	11/9	9/11	7
Ito − 2016	PSM	2011–2013	Japan	27	27	69 (66–72)	71 (68–74)	16/11	15/12	8
Lai-2016	Retrospective	2005–2010	China	33	28	62.8 ± 11.3	56.5 ± 12.6	29/4	24/4	7
Yazici-2016	Retrospective	2000–2014	USA	41	41	73.7 ± 5	72.6 ± 6.7	24/17	25/16	8
Yamashita-2018	Retrospective	2000–2016	Japan	62	38	66.5 ± 9.5	66.9 ± 9.1	40/22	25/13	8
Tsukamoto-2019	Retrospective	2000–2017	Japan	94	77	67.4 ± 8.1	65.2 ± 10.2	51/43	53/24	7
Sandro-2019	PSM	2006–2016	Italy	50	50	67 (56, 76)	68 (62, 76)	37/13	33/17	8
Chong-2019	PSM	2014–2016	China	59	59	59.3 ± 11	57.7 ± 10.5	46/13	46/13	7
Pan-2019	PSM	2014–2016	China	236	118	56.00 (45–64)	53.00 (45.2–61)	206/30	101/17	7
Lee-2020	PSM	2014–2016	Korea	118	118	60.5 ± 10.3	59.5 ± 8.7	88/30	91/27	7
Ogiso-2020	Retrospective	2011–2016	Japan	136	85	73 (47–87)	69 (46–88)	98/38	62/23	8

LH laparoscopic hepatectomy, RFA radiofrequency ablation, M/F male/female, PSM propensity-score matching, NOS Newcastle-Ottawa Scale

**Table 2:** Summary results of the meta-analyses

Outcomes of interest	Studies, *n*	RFA	LH	WMD/OR (95%CI)	*P* value	Heterogeneity			
						X2	df	I^2^,%	*P* value
Opertive outvomes									
Operative time(min)	5	308	262	−199.26(−163.67,−74.84)	< 0.00001	70.33	4	94	< 0.00001
blood loss	5	308	262	−232.5(−300.55,−164.45)	< 0.00001	10.38	4	61	0.03
tumor size	12	910	682	−0.2(−0.38,−0.02)	0.03	140.35	11	92	< 0.00001
**Postoperative outcomes**									
overall complication rates	10	768	538	0.5(0.33,0.76)	0.001	7.29	9	0	0.61
Length of stay	10	826	596	−3.34(−4.49,−2.18)	< 0.00001	984.54	9	99	< 0.00001
**Oncological outcomes**									
local recurrence rate	6	316	272	3.9(2.25,6.77)	< 0.00001	5.38	5	7	0.37
1-year overall survival	10	800	572	0.65(0.31,1.35)	0.24	17.51	9	49	0.04
3-year overall survival	11	850	622	0.79(0.48,1.27)	0.33	29.32	10	66	0.001
5-year overall survival	9	581	476	0.68(0.51,0.9)	0.008	11.05	8	28	0.2
1-year recurrence-free survival	11	908	695	0.38(0.27,0.54)	< 0.00001	18.87	10	47	0.04
3-year recurrence-free survival	11	908	695	0.49(0.3,0.8)	0.005	37.6	10	74	< 0.001
5-year recurrence-free survival	9	639	549	0.51(0.23,1.11)	0.09	40.39	8	80	< 0.00001

LH laparoscopic hepatectomy, RFA radiofrequency ablation, MD mean difference, OR odds ratio, CI confidence interval

### Operative outcomes

#### Operative time

**Fig. 2:**
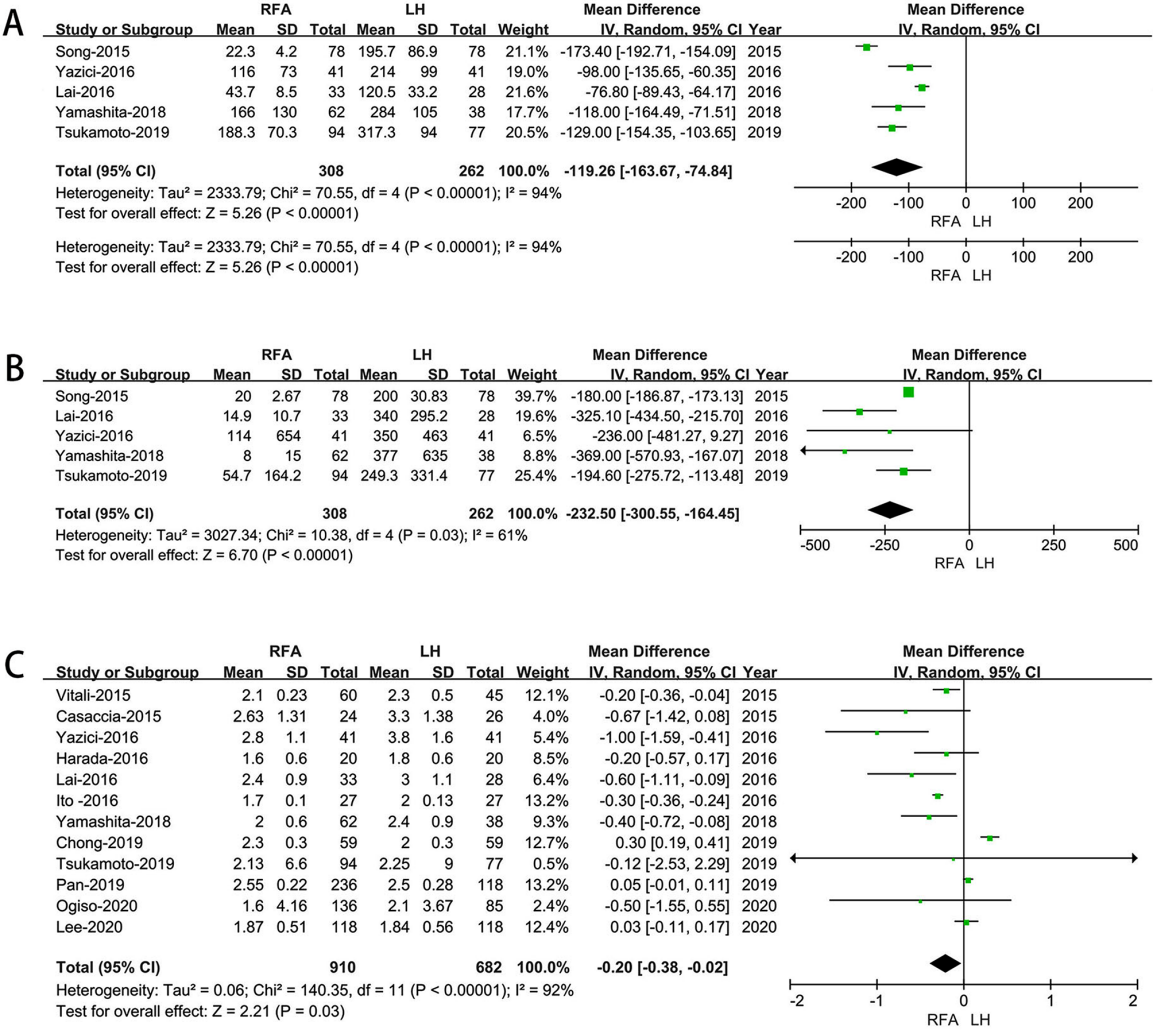
Forest plot of comparison of LH versus RFA for operative outcomes. **A**: Forest plot for Operative time; **B**: Forest plot for Blood loss; **C**:Forest plot for Tumor size

There were five studies [19, 23–26] which covered 570 patients (262 underwent LH whereas 308 underwent RFA) reported operative times. It was found that the LH group had a longer operative time (MD: -119.26 min; 95% CI: -163.67 to -74.84; *p* < 0.00001). Heterogeneity of the data was high (I^2^ = 94%) and analyzed in REM (Fig. 2A).

#### Blood loss

As with the surgery time, a total of five literature had reported on the amount of bleeding [19, 23–26]. As shown in Fig. 2B, the pooled data revealed a significant reduction in blood loss in the RFA group(MD: −232.5 ml; 95% CI: −300.55 to – 164.45; *p*<0.00001).

#### Tumor size

Tumor size data was available in 12 studies [18, 20–26, 28–30]. The meta-analysis suggested tumor size was smaller in RFA group (MD: -0.20; 95% CI: -0. 38 to -0.02; *P* = 0.03).(Fig. 2C).

### Postoperative outcomes

#### Overall complication rates

**Fig. 3:**
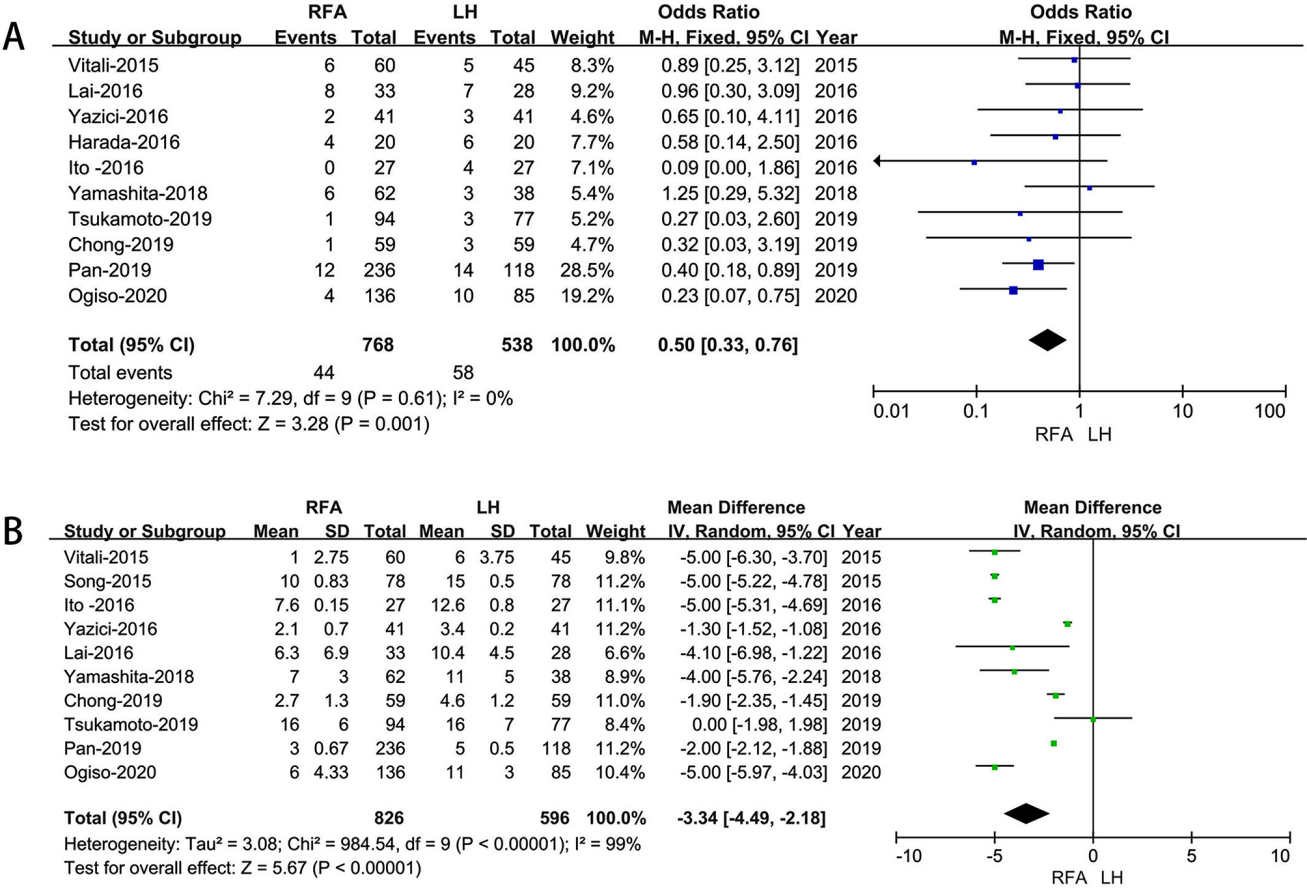
Forest plot of comparison of LH versus RFA for Postoperative outcomes. **A**: Forest plot for overall complication rates; **B**: length of stay

Ten studies [20–26, 28–30]that encompassed 1,306 patients (538 and 768underwent LH and RFA, respectively) reported the overall complications, and the present analysis shows LH group has lower overall complication rate (OR: 0.50; 95% CI 0.33 to 0.76; *p* = 0.001). (Fig. 3A).

#### Hospital stay

Based on eight studies [19, 20, 22–26, 28–30] which included 1,422 HCC patients, the meta-analysis demonstrates that RFA treated HCC had a shorter hospital stay when compared to LH treated HCC. (MD = − 3.34; 95% CI – 4.49 to – 2.18; *p*<0.00001), (Fig. 3B).

### Oncological outcomes

#### Local recurrence rate

**Fig. 4:**
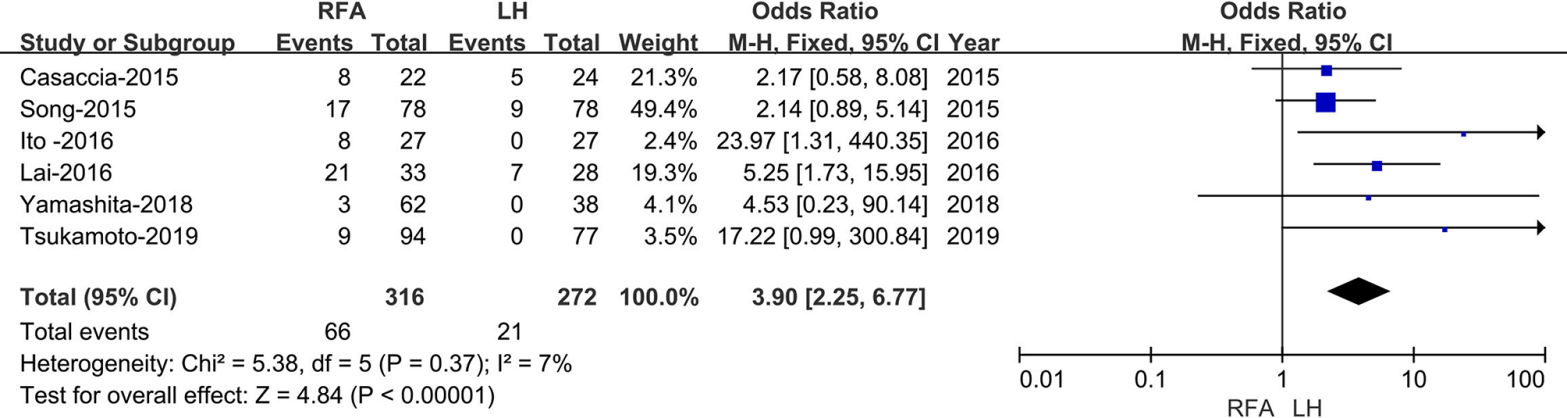
Forest plot of comparison of LH versus RFA for local recurrence rate

Nine studies provided data regarding the local recurrence rate [19–21, 23–28]. The results showed that the LH group had a lower local recurrence rate (OR:3.90; 95% CI 2.25–6.77; *p* < 0.00001), with low heterogeneity (I^2^ = 7%) as shown in the FEM (Fig. 4).

#### Overall survival

**Fig. 5:**
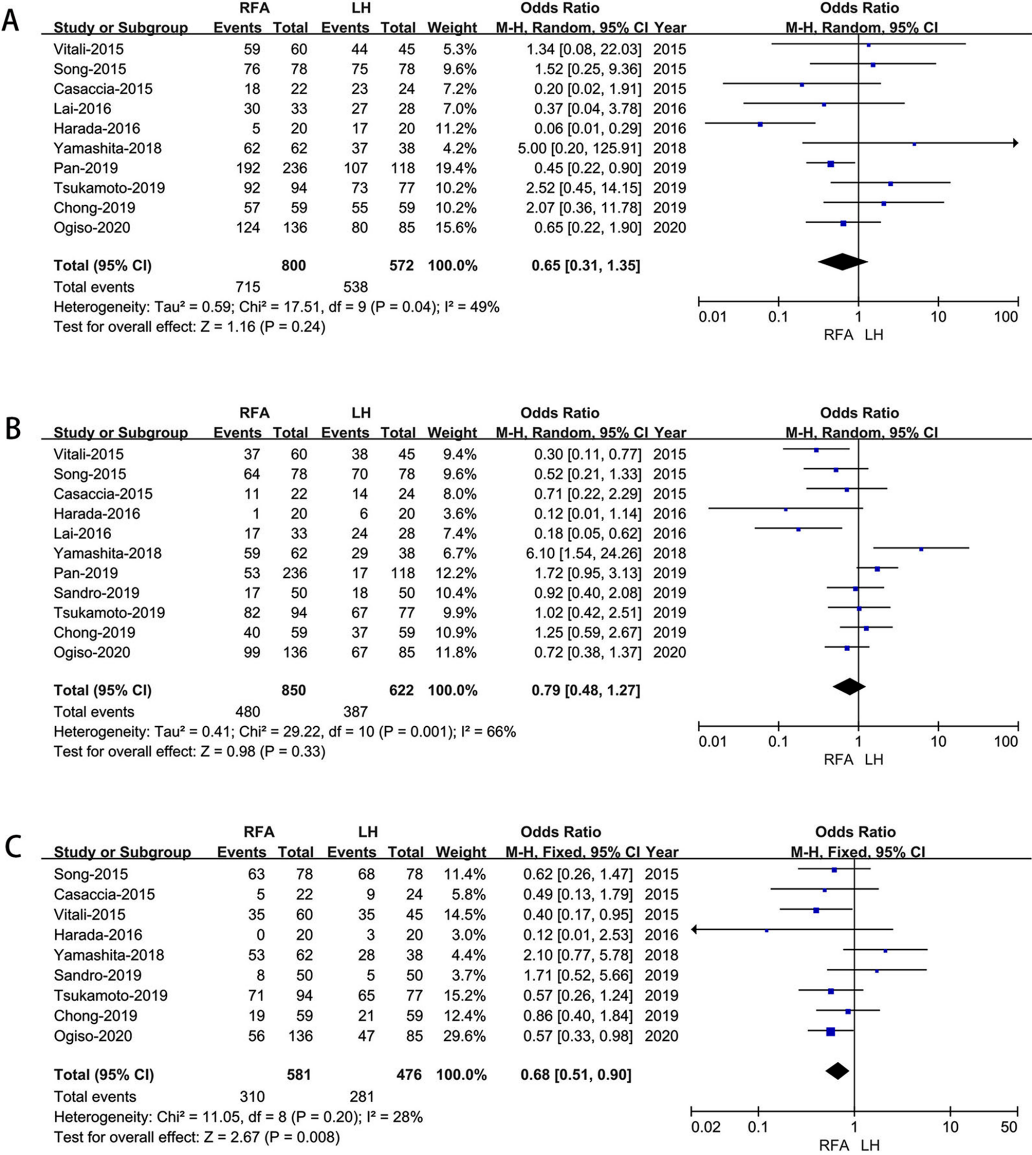
Forest plot of comparison of LH versus RFA for long‑term oncological outcomes **A**: 1-year overall survival time; **B**: Forest plot for 3-year survival time; **C**: Forest plot for 5-year survival time

Ten studies [18–21, 23, 25, 26, 28, 29, 31]assessed 1-year overall survival.The results showed no difference in the 1-year overall survival rate between the two groups(OR: 0.65; 95% CI 0.31 to 1.35; *p* = 0.24), with moderate heterogeneity (I^2^ = 49%) in the REM (Fig. 5A). Ten studies [18–21, 23, 25, 26, 28, 29, 31]assessed 3-year overall survival, the result of meta-analysis revealed no difference in 3-year overall survival (OR: 0.79; 95% CI 0.48 to1.27; *p* = 0.33), with high heterogeneity (I^2^ = 66%) in the REM (Fig. 5B). Nine studies [18–21, 23, 25, 26, 28, 31] assessed 5-year overall survival.The results showed that the LH group had a higher overall 5-year survival rate (OR: 0.68; 95% CI 0.51 to 0.90; *p* = 0.008), with low heterogeneity (I^2^ = 28%) in the FEM (Fig. 5C).

#### Recurrence-free survival

**Fig. 6:**
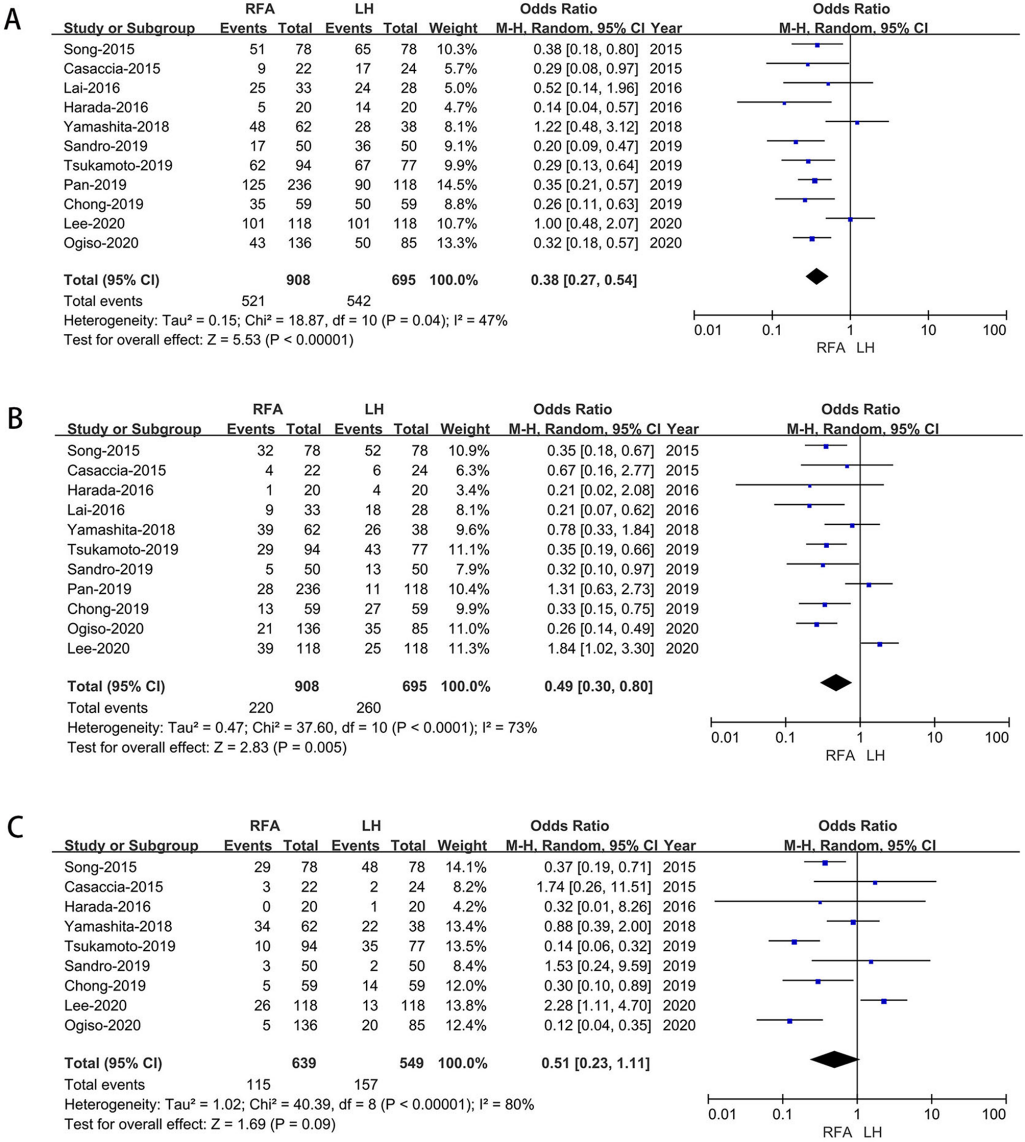
Forest plot of comparison of LH versus RFA for long‑term oncological outcomes. **A**: Forest plot for 1-year overall survival time; **B**: Forest plot for 3-year survival time; **C**: Forest plot for 5-year survival time

Eleven studies [18, 19, 21, 23, 25–30] that included 1,603 patients (695 who underwent LH and 908 who underwent RFA) assessed 1-year and 3year RFS rate, the result of meta-analysis revealed RFA has higher1-year and 3-year RFS rate (*p*<0.00001,*p* = 0.005,respectively) (Fig. 6A-B). Nine studies [18, 19, 21, 25–28, 30, 31] assessed 5-year RFS rate, the result of meta-analysis revealed no difference in 5-year RFS rate between LH and RFA (OR: 0.51; 95% CI 0.23 to 1.11; *p* = 0.09), with low heterogeneity (I^2^ = 80%) in the REM (Fig. 6C).

#### Publication bias

Using Begg’s funnel plot, publication bias was investigated. For overall complications and 5-year survival, all studies lie within 95% CIs, indicating no publication bias. (Figure S2)

## Discussion

Minimally invasive surgery and specialization of minimally invasive surgery are the development trend of modern surgery. Due to the rapid development of surgical technology and surgical instruments, as well as the rapid progress of imaging technology, enables surgeons to accurately judge the location, size and whether the surrounding vessels and organs are violated before operation, which greatly promotes the development of minimally invasive surgery of the liver. As Reich et al. performed the first case of LH in 1991, laparoscopic technique gained popularity due to its magnifying effect and wider visual field. At present, LH has been proved to be safe and similar long-term oncological outcomes over traditional surgery, but the surgical procedure is difficult and time-consuming [32–34]. The results of previous studies of the outcomes of LH to RFA have been inconsistent. Throughout this meta-analysis, the latest studies from 2015 to 2023 were included to compare the safety and efficacy of LH compared to RFA in the treatment of HCC. Despite the absence of any randomized controlled trials (RCTs), the majority of the studies included were propensity score matching (PSM) studies and demonstrated a relatively high quality based on the NOS assessment, as indicated in Table 1.

In the past few years, radiofrequency ablation and laparoscopic liver resection have been popular choices for the treatment of liver cancer. In order to better understand the effectiveness and advantages and disadvantages of these two methods, we conducted a meta-analysis and conducted a comprehensive evaluation of relevant research. Through collecting and analyzing a large amount of research data, we found that both radiofrequency ablation and laparoscopic liver resection have certain therapeutic effects in the treatment of liver cancer. However, there are differences between these two methods in certain aspects.

Firstly, from the perspective of surgical trauma and recovery, radiofrequency ablation has the advantages of minimally invasive and fast recovery. Through local puncture to destroy liver cancer tissue, radiofrequency ablation has less trauma to patients and faster postoperative recovery. Although laparoscopic liver resection is also a minimally invasive surgery, it requires the establishment of a small incision in the abdomen, which can cause relatively greater trauma to the patient.

Secondly, in terms of tumor control and recurrence rate, laparoscopic liver resection has a wider resection range and lower recurrence rate. For larger liver cancer or multiple liver cancer, laparoscopic liver resection can more thoroughly remove tumor tissue and reduce the risk of recurrence. Radiofrequency ablation is mainly used for the treatment of small liver cancer or unresectable liver cancer, and its efficacy may be limited for larger or multiple liver cancer.

Two previous meta-analyses comparing perioperative and oncologic outcomes of LH to RFA were published. However, the sample size of both studies was relatively small [12, 13]. Jin et al’s [8] study only focus on perioperative outcomes between LH and RFA. Seven articles were included in Jin et al’s study, and they claim that seven studies are RCTs. After carefully reading these 7 articles, we found that 3 articles were in Chinese and none of them were RCTs. Their results are not credible. Li et al’s [13] meta-analysis covered 1,570 HCC participants from 10 retrospective studies and focused on and longterm survival outcomes. They used the data before the PSM to analysis to get a sufficient sample size and 3 of those studies are conference abstracts. They found that LH was associated with longer 1-,3-, and 5-year overall survival time, better 1-year and 3-year DFS rate, lower local recurrence rate and higher complications.

Comparing with their results, our study included some recent studies [28–30], and excluded conference abstracts. The meta-analysis showed LH has a longer hospitalization, more blood loss and longer operative time but a lower local recurrence rate than RFA, which was consistent with the study of Jin et al. The main cause of longer operation time of LH is hemostasis and suture were performed on the liver section.

Local recurrence rate is an important malignancy prognosis factor for HCC [35]. The present meta-analysis revealed that LH has a lower rate of local recurrence than RFA. This difference may be explained by patients with HCC in early stage were selected to perform LH and RFA for larger or irregular tumor may exist three-dimensional leakage phenomenon, resulting in residual lesions, and the scope of thermal ablation is limited. From a clinical point of view, the results of this meta-analysis show that LH has higher 1-and 3- RFS rate, suggesting that LH has better tumor radical effect than RFA.

According to our knowledge, no RCTs have been conducted in patients with HCC that compare long-term survival between LH and RFA. The largest overall survival outcomes data of LH and RFA in the treatment of HCC comes from Korea. Lee et al. reported 566 patients with HCC underwent LH or RFA (251 underwent LH and 315 underwent RFA) and revealed that nonsignificant difference in 1-,2-and 3-year overall survival time in the two groups (100, 99.5, and 97.9% vs. 99.0, 98.3, and 97.2% respectively, *p* = 0.16) [30]. But Pan et al. compared the survival data after PSM of 354 patients with HCC (118 underwent LH and 236 underwent RFA) from China and suggested that LH and RFA can achieved a median overall survival of 25.6 and 23.4 months respectively (*p* = 0.034) and LH has better 1-,2-and 3-year overall survival rate than RFA (97.3, 97.3 and 91.0% vs. 99.5, 87.0 and 79.0%, respectively, *p* = 0.0034) [29]. Although our meta-analysis revealed no significant difference in 1- and 3-year overall survival rate (OR: 0.65; 95% CI 0.31 to 1.35; *p* = 0.24; OR: 0.79; 95% CI 0.48 to1.27; *p* = 0.33, respectively), LH has better 5-year overall survival rate (OR: 0.68; 95% CI 0.51 to 0.90; *p* = 0.008). Based on the pooled data, LH does not appear to be inferior to RFA from an oncological perspective and is actually able to achieve a superior oncologic outcome when compared to RFA in some ways.

In the era of minimally invasive liver resection, robotic surgery is increasingly being explored for its safety and effectiveness in liver resection due to its inherent advantages, including flexible mechanical arms with 360-degree range of motion, strong stability, precision in operation, 3D magnified vision, and improved comfort for the surgeon [36]. Charing et al.‘s study found that robot-assisted liver resection has a lower conversion rate to open surgery and reduces postoperative hospital stay compared to laparoscopic liver resection. Once the learning curve is overcome, the conversion rates are similar [37]. Several studies comparing robot-assisted liver resection with traditional open liver resection have found that while the operative time for robot-assisted procedures is longer, the postoperative hospital stay is significantly shorter, and the incidence of severe complications, such as liver failure, is markedly reduced [36, 38]. Nicholas et al. observed a lower 45-day readmission rate for patients undergoing robotic liver resection [39]. Aziz et al. found that the 6-month readmission rate following robotic surgery was lower compared to traditional surgery [40]. It is noteworthy that Zhu et al. found no significant difference in long-term outcomes, including 5-year DFS and OS, among patients with BCLC 0-A stage HCC undergoing robotic-assisted liver resection, laparoscopic liver resection, or traditional open surgery [41].

Despite the significant minimally invasive advantages of radiofrequency ablation (RFA) for liver cancer, there are challenges in safely ablating tumors that are difficult to reach using conventional ultrasound or CT guidance. To further advance the minimally invasive treatment of liver cancer, some researchers are exploring the combination of robotic-assisted navigation systems with RFA [42]. This integration holds promise for enabling RFA of tumors in difficult-to-reach locations under robotic guidance. Additionally, due to the remote control capabilities of robots, this approach could facilitate telemedicine support. Although research in this area is still limited, it is worth exploring as robotic assistance has the potential to revolutionize the future of minimally invasive RFA.

The most advantageous areas for RFA in the treatment of HCC are early HCC (diameter ≤ 3.0 cm) and very early hepatocellular carcinoma (diameter ≤ 2.0 cm) [43]. A large amount of high-level evidence-based evidence indicates that the curative effect of RFA is not significantly different from that of liver resection and liver transplantation for these two types of HCC, and in most cases, it can be the first choice.

Medium volume HCC (diameter 3.1–5.0 cm) is not a good indication for RFA treatment. Its main pathological feature is that the surrounding microvascular invasion (MVI) is relatively broad, and its pathological volume is much larger than the volume of the main tumor visible on imaging. It is difficult to achieve pathological complete ablation using RFA alone. In principle, the preferred treatment for medium volume HCC is liver resection. For those who cannot or are unwilling to undergo liver resection, RFA can be selectively applied. Generally speaking, the higher the degree of differentiation of HCC, the more complete the capsule, and the better the efficacy of RFA. Microwave ablation, like radiofrequency ablation, is a minimally invasive therapy for liver cancer that was introduced around the same time. The principle is to rapidly and uniformly heat tissues through electromagnetic energy. Initially, people were not clear about the differences between the two, but with clinical applications, multiple studies comparing their efficacy have gradually been published, but the results are slightly different. Most studies indicate no significant difference in disease-free survival (DFS) and overall survival (OS) between the two ablation methods [44, 45].But a recent meta-analysis showed that microwave ablation can lead to better prognosis and fewer complications [46]. Compared to radiofrequency ablation, microwave ablation has a shorter heating time, a larger ablation area, and a smaller heat sink effect.Consequently, some studies recommend prioritizing microwave ablation for tumors larger than 3 cm or those located adjacent to major blood vessels [47].

In addition, the combination of transcatheter hepatic artery embolization chemotherapy (TACE)/hepatic artery embolization (TAE), with a safety margin of 1.0 cm and consolidation repeat ablation, is an important strategy to improve the ablation efficiency of medium volume HCC and achieve pathological complete ablation.

Solitary large HCC has a huge volume, and single RFA treatment is difficult to achieve complete ablation. The preferred treatment method is liver resection. For patients who cannot be removed, RFA can be considered in combination with TACE/TAE.

Recurrent hepatocellular carcinoma is a good indication for RFA[48]. The compliance of postoperative follow-up in patients with hepatocellular carcinoma is usually good, and recurrent HCC lesions are usually small, making it easy to achieve complete ablation using RFA. Research has shown that there is no significant difference in the efficacy between repeat liver resection and RFA for patients with recurrent HCC. In summary, both radiofrequency ablation and laparoscopic liver resection have certain therapeutic effects in the treatment of liver cancer. However, there are differences between these two methods in terms of surgical trauma, tumor control, recurrence rate, and risk of complications. When selecting treatment methods, comprehensive consideration should be given to factors such as the patient’s specific condition, tumor size, location, and the doctor’s experience and technical level.

It is important to consider the limitations of the present meta-analysis, even though it includes several PSM studies for a more credible conclusion. First, it is possible that selection bias was caused by the majority of the studies included were retrospective studies and no RCTs was include. Moreover, although all the trials described the distribution of the included cases and the factors affecting the prognosis, it was difficult to achieve the complete matching of the baseline data between the two groups, and the statistical treatment of reducing bias was not used in the statistical analysis stage, and the sample size of some studies was small, so there might be some occurrence bias. It is also important to emphasize that there are insufficient studies reporting long-term survival outcomes. Hence, to further evaluate LH’s safety and efficacy in patients with HCC, large-scale and RCT studies with long-term outcomes will be needed .

## Conclusions

Overall, our study showed LH was safe, feasible, and technically feasible for HCC patients, providing better 5-year OS and 1-,3-year RFS. Both LH and RFA are the radical minimally invasive treatment for early-stage HCC. Patients with smaller tumor size should choose LH to resect tumor tissue completely to reduce recurrence and obtain longer RFS rate and overall survival. For young patients with HCC, surgical resection should be preferred to achieve better curative effect. However, the older patients can choose the two ways. In view of RFA has advantage of less trauma and shorter operation time, for the elderly and weak patients, or patients with high risk of surgery combined with other diseases, can choose RFA first.

### Electronic supplementary material

Below is the link to the electronic supplementary material.

Supplementary Material 1

Supplementary Material 2

## Data Availability

No datasets were generated or analysed during the current study.

## Abbreviations

LH: Laparoscopic hepatectomy
RFA: Radiofrequency ablation
HCC: Hepatocellular carcinoma
OS: Overall survival
RFS: Recurrence-free survival
HR: Hazard ratio
NOS: Newcastle-Ottawa Scale
OR: Odds ratio
MD: Mean differences
FEM: Fixed-effects model
REM: Random-effects model
MVI: Microvascular invasion
CI: Confidence interval
