# Supplementary figures and images for: Radiofrequency ablation versus laparoscopic hepatectomy for hepatocellular carcinoma: a systematic review and meta-analysis
Source: World J Surg Oncol. 2024 Jul 24;22:188. doi: 10.1186/s12957-024-03473-8 (PMC11267765; doi:10.1186/s12957-024-03473-8)

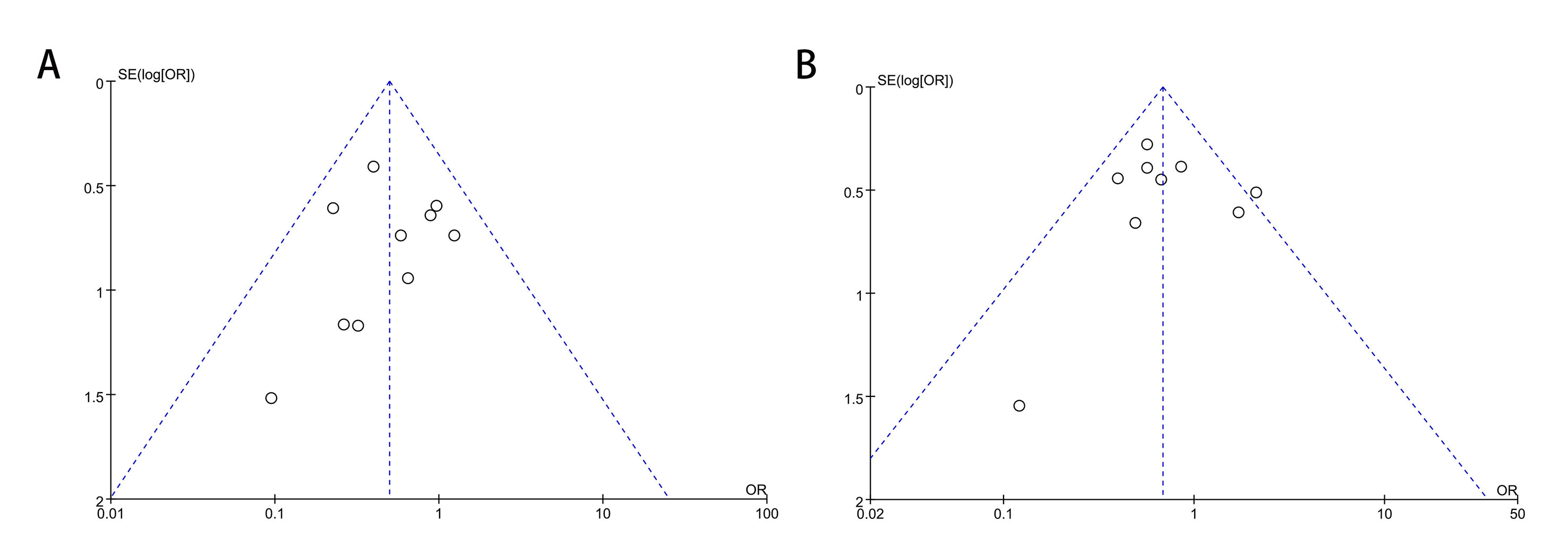

Supplement: Supplementary file 1 — Supplementary Material 1 [file 12957_2024_3473_MOESM1_ESM.jpeg]

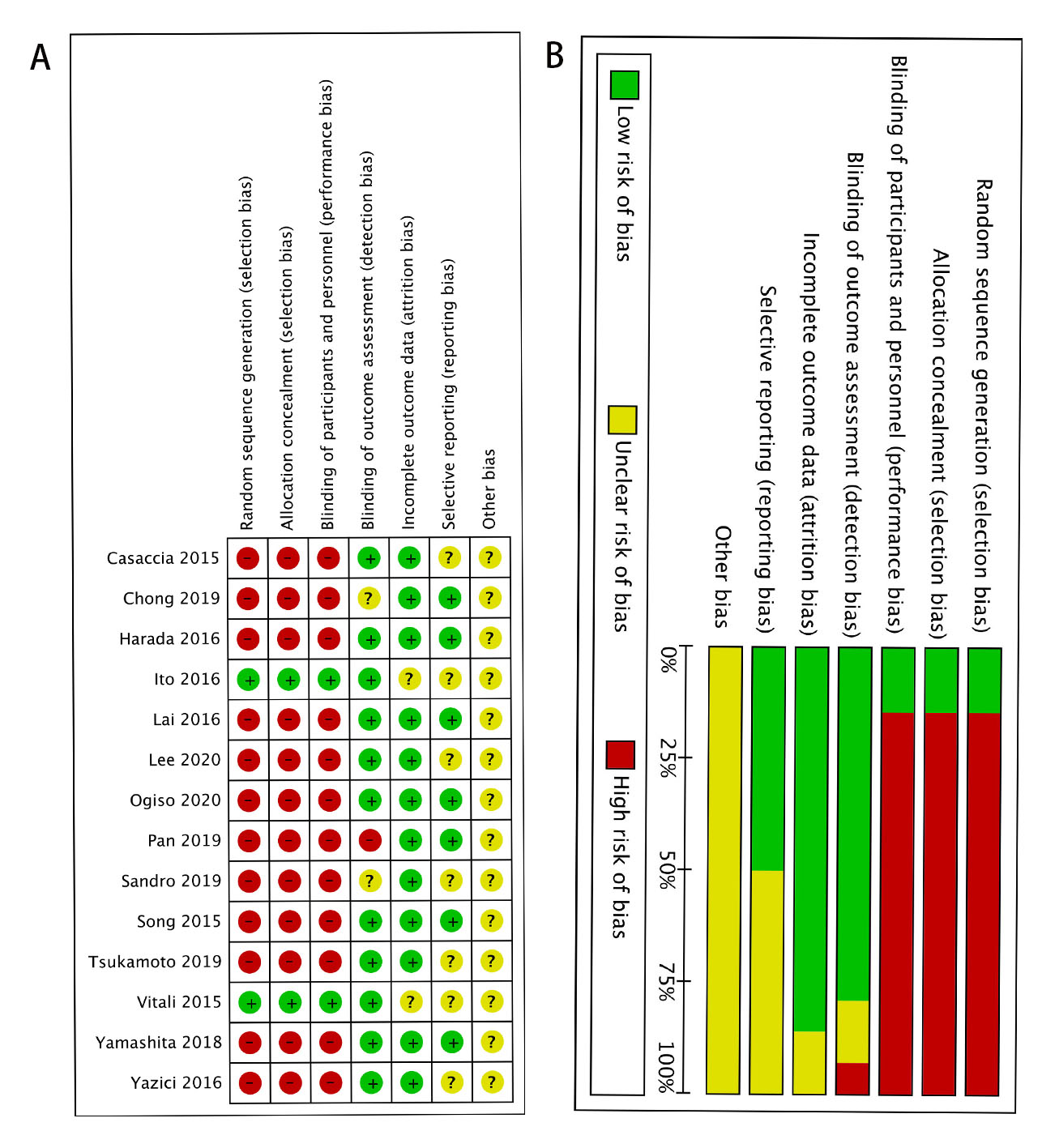

Supplement: Supplementary file 2 — Supplementary Material 2 [file 12957_2024_3473_MOESM2_ESM.jpeg]
